# Optimal features selection in the high dimensional data based on robust technique: Application to different health database

**DOI:** 10.1016/j.heliyon.2024.e37241

**Published:** 2024-09-02

**Authors:** Ibrar Hussain, Moiz Qureshi, Muhammad Ismail, Hasnain Iftikhar, Justyna Zywiołek, Javier Linkolk López-Gonzales

**Affiliations:** a Department of Statistics Abdul Wali Khan University Mardan, Pakistan; b Govt Boys Degree College Tandojam, Hyderabad, Sindh, Pakistan; c College of Statistical Sciences, University of the Punjab, Lahore, Pakistan; d Department of Statistics, Quaid-i-Azam University, 45320, Islamabad, Pakistan; e Faculty of Management, Czestochowa University of Technology, Czestochowa, 42-200, Poland; f Escuela de Posgrado, Universidad Peruana Unión, Lima, Peru

**Keywords:** High-dimensional data, Optimizing gene selection, Mood median test, Single noise ratio score, Machine learning models, Hybrid technique

## Abstract

Bio-informatics and gene expression analysis face major hurdles when dealing with high-dimensional data, where the number of variables or genes much outweighs the number of samples. These difficulties are exacerbated, particularly in microarray data processing, by redundant genes that do not significantly contribute to the response variable. To address this issue, gene selection emerges as a feasible method for identifying the most important genes, hence reducing the generalization error of classification algorithms. This paper introduces a new hybrid approach for gene selection by combining the Signal-to-Noise Ratio (SNR) score with the robust Mood median test. The Mood median test is beneficial for reducing the impact of outliers in non-normal or skewed data since it may successfully identify genes with significant changes across groups. The SNR score measures the significance of a gene's classification by comparing the gap between class means and within-class variability. By integrating both of these approaches, the suggested approach aims to find genes that are significant for classification tasks. The major objective of this study is to evaluate the effectiveness of this combination approach in choosing the optimal genes. A significant P-value is consistently identified for each gene using the Mood median test and the SNR score. By dividing the SNR value of each gene by its significant P-value, the Md score is calculated. Genes with a high signal-to-noise ratio (SNR) have been considered favorable due to their minimal noise influence and significant classification importance. To verify the effectiveness of the selected genes, the study utilizes two dependable classification techniques: Random Forest and K-Nearest Neighbors (KNN). These algorithms were chosen due to their track record of successfully completing categorization-related tasks. The performance of the selected genes is evaluated using two metrics: error reduction and classification accuracy. These metrics offer an in-depth assessment of how well the selected genes improve classification accuracy and consistency. According to the findings, the hybrid approach put out here outperforms conventional gene selection methods in high-dimensional datasets and has lower classification error rates. There are considerable improvements in classification accuracy and error reduction when specific genes are exposed to the Random Forest and KNN classifiers. The outcomes demonstrate how this hybrid technique might be a helpful tool to improve gene selection processes in bioinformatics.

## 1 Introduction

In many different sectors, data-driven decision-making relies heavily on knowledge discovery and data mining. They make it possible for businesses and individuals to use data to make wise decisions, obtain a competitive edge, and deepen their comprehension of intricate systems [1,2]. Enormous amounts of data are being generated across diverse fields, and this growth is ongoing, marked by expansions in scale, intricacy, and the number of dimensions. A dataset characterized by high dimensionality possesses many features; however, only a small number of these features directly correlate with tasks in data mining and machine learning [3,4]. Hence, these challenges posed by data complexity present significant hurdles when attempting to uncover potentially valuable and comprehensible patterns or insights in nearly every data mining endeavor. Furthermore, navigating within high-dimensional data exacerbates the intricacies of knowledge discovery and pattern classification due to the abundance of redundant and extraneous features [5,6]. Mitigating this issue involves dimensionality reduction, wherein high-dimensional datasets are transformed into lower-dimensional ones by filtering out redundant and noisy information. This technique serves as a solution to address the issue and is commonly known as dimensionality reduction [7,8]. This study [9] suggested using choosing features and hyper-parameter optimization as approaches for short-term traffic predictions.

In dimensional data, the number of features (genes) in gene expression datasets may be significantly more than the number of observations (samples). This high dimensionality poses challenges for conventional estimation methods. Dimensionality reduction must be performed to handle high-dimensional datasets with limited observations to get the optimal genes in the high-dimensional datasets [10,11]. In order to calculate semantic similarity, this study [12] suggests two new concepts, (SCSP) and (SCSN). Next, the Pseudo-label generator estimates each sample's label based on these ideas as well as the quantity of sentiment words.

Feature Selection approach reduces the dimension in high dimensional datasets, and also picks up a small part of relevant and revelatory genes or features from the entire feature space [13]. To improve the efficiency of machine learning activities, feature selection has drawn a lot of attention. The fuzzy rough set is one of the most often used feature selection techniques because of its ability to deal with ambiguity [14]. This study looks at how best to schedule (BESSs) to minimize the negative effects that charging electric vehicles (EVs) has on power distribution networks. To address this issue, a (DDPG) and evolutionary algorithm combination has been used [15].

Feature Extraction Unlike feature selection, feature extraction reduces dimensionality by transforming the original set of features into a smaller number of new features. These new features are a combination of the original features and are derived through various mathematical techniques such as principal component analysis (PCA) [16,17]. Both feature selection and feature extraction have their strengths and weaknesses, and the choice of method depends on the specific data set and the goals of the analysis. It's important to note that proper feature selection or extraction can lead to improved model performance, reduced computational complexity, and better interpretation of results, especially in scenarios where high-dimensional data is encountered with limited observations. This article [18] describes the methodical development of an ultra-local model (ULM) controller for (LFC) of a (MMG) using an (ESO). SNR which describes channel quality in communication systems, is a crucial statistic. Many signal processing methods or approaches, including signal identification, quality control, tub-compress, stenography reconciling transmission, require a priori information. SNR statistic or estimator is typically divided into two categories. The first case in the SNR is a data-aid (DA) estimator and a non-data-aid (NDA) statistic. These categories are based on the amount of information that is currently accessible for a received signal. In inherited information is known to the obtain side, a DA estimate, such as the maximum likelihood (ML) SNR estimator [19], or Squared signal-to-noise variance (SNV) estimator [20], can typically be employed. The split-symbol moment estimator (SSME) [21], and the second and fourth moment (M2M4) estimator [22]. Recent studies demonstrated that gene expression patterns produced using microarray technology have a significant impact on cancer disease. Microarrays offer details on the degree to which the genes represented on the array are expressed. Some studies have looked at associations between a gene or a group of genes' levels of expression and clinically significant subcategories of particular tumor subtypes [23].

Both the signal-to-variation ratio (SVR) estimator and the split-symbol moment estimator (SSME) [24] may calculate the SNR simply from the unknown, information-bearing fraction of the received signals. Although DA estimators may perform better, NDA estimators do not interfere with the channel's throughput and require less a priori data. NDA estimators are hence more useful, particularly in non-cooperative circumstances a more scientific method for detecting cancer.

The results gathered from observation suggest that accurate molecular staging and sub-classification of various tumor types may be feasible, allowing prediction and management of patients. It has been studied by various researchers that subgroups of cancers that differ according to the types of tumor and histologist subclasses of tumors, exist among carcinogenic patients, using microarray technologies while evaluating cancer cells [25,26]. In an independent Component Subspace Weighted-SNR Feature Selection for NB Data Classification from Micro-arrays, the primary basis for 246 cells is their morphological behavior. On the other hand, it can frequently be quite challenging to distinguish clearly between various cancer kinds based solely on appearances. As a result, the micro-array technique may offer a more precise method of cancer diagnosis [27,28]. Thousands of genes' patterns of expression can be ascertained using microarrays, which provide an effective technique for collecting data [29]. The pattern of mRNA expression in various organs in healthy and diseased states may help identify the genes and environmental factors that can cause disease. RNA was first extracted from a tissue sample or probe as the first step in the usual micro-array experiment. In the end, fluorescent (usually red) DNA is produced after the mRNA has been marked with fluorescent nucleotides. The sample is then treated with a reference DNA that has undergone similar processing (usually in green). Fluorescent sequences in the labeled probe-reference mix are then combined and applied to the surface of DNA micro-arrays, enabling the DNA adherent to the glass slide to be bound to the fluorescent sequences in the probe-reference mix. The allure of DNA that has been designated [30,31]. The amount to which the sequences in the mixture (probe-reference) complement the DNA attached to the slide determines the attraction of labeled c DNA from the probe and reference for a specific area on the micro-array. Hybridization is the process through which a nucleotide sequence on a strand of DNA perfectly complements a DNA sequence attached to the slide. The most important component of microarray technology is hybridization [32]. A laser is used to excite the filled microarray, and the resulting fluorescence at each location on the microarray is then measured [33,34]. This study makes use of the recently disclosed (CPC) fractional operator in conjunction with extended Fourier and Fick's equations [35,36]. In this work [37,38], the proportional and Caputo operators are combined to create a fractional operator called the constant proportional Caputo, which is the first of its kind. The investigation of the unsteady Casson flow model was done using this newly constructed operator to show its dynamic properties. The goal of the work [39,40] is to create and improve a Brinkman flow model for a channel consisting of two parallel vertical plates. This is accomplished by applying the more sophisticated and comprehensive definition of the Prabhakar operator to the constitutive relations for mass and heat fluxes.

### 1.1 Objective of the study

The presence of redundant or unnecessary variables (genes) in high-dimensional data analysis is a significant problem that may reduce the effectiveness of classification algorithms and confuse the study. In order to make gene selection for classification algorithms more successful, this study recommends a hybrid strategy that combines the Mood Median test with the SNR score.

The Signal-to-Noise Ratio (SNR) score with the Mood median test to present a novel way to identify genes relevant for classification tasks. Because it is robust to the impact of outliers or tainted observations in the data, the mood median test is the best option when dealing with irregular observations. The genes that are most crucial for differentiated classes are found using these statistical methods. The effectiveness of the selected genes is then evaluated using two powerful classification algorithms, Random Forest and K-Nearest Neighbors (KNN). These methods were chosen due to their excellent classification task performance, providing a comprehensive assessment of classification accuracy and error reduction. The classification of genes using this approach is guaranteed to be accurate and consistent.

The rest of the article is shaped as follows. In section 2, this study demonstrates material and methods applied to the data additionally, section 3 discusses the results of the proposed and existing methods on gene data set, and finally, a conclusion and recommendations are given in section 4.

## 2 Method and materials

In this section, this study discusses all the models and methods considered in the current research work. The details about all models and techniques are the following.

### 2.1 Random Forest

The Random Forest (R.F.) algorithm has gained popularity in the field of life sciences due to its compatibility with datasets where the number of predictors (p) greatly exceeds the number of observations (n) [[41], [42], [43], [44]]. It exhibits resilience to substantial levels of noise, minimizing the impact of such noise on its performance. Additionally, this algorithm necessitates minimal parameter adjustment, and there's no requirement for transforming predictors before its application. One of the notable advantages of Random Forest is its inherent capability to provide a measure of feature importance. This assessment directly elucidates a feature's contribution within all interactions encompassed by the model, even encompassing subtle and intricate relationships. This inclusive consideration of weak and multivariate interactions further enhances the algorithm's effectiveness. Considering these attributes, Random Forest holds significant promise as a classification algorithm for tasks involving gene selection, offering a robust and insightful approach within this context. It enhances the accuracy and robustness of predictions by aggregating the results and producing reliable outcomes, making it suitable for various tasks in classification [[45], [46], [47], [48]].

### 2.2 K-Nearest Neighbors

The K-Nearest Neighbors (KNN) algorithm is the supervised learning technique. It is used for classification and regression problems. The data is divided into two phases the first phase is included the training data and the second phase involves testing data the training data is the labeling they have the responses variable in the data while the testing data have to check out the permanence of the algorithm. In the classification problem, the KNN algorithm used the majority of voting and the regression condition used the average value.

During its learning phase, the KNN algorithm doesn't abstract any insights from the training data. The process of generalization is deferred until the classification stage. The classification process with KNN involves identifying the nearest neighbor in the instance space and assigning the unknown instance the same class label as its nearest known neighbor. This methodology is commonly referred to as a nearest neighbor classifier. Due to its high sensitivity to local variations, nearest neighbor classifiers are susceptible to noise present in the training data [49,50].

To enhance the robustness of the model, a technique involves considering a value of "k" greater than 1, where the class label is determined through a majority vote among the k neighbors. When k equals 1, the object is simply assigned to the class of its closest neighbor. Larger values of k result in a smoother and less locally sensitive classification function. To determine closeness, various distance measures are typically used. These include Euclidean distance, Manhattan distance, and Minkowski distance for continuous variables. For categorical variables, the Hamming distance is the appropriate choice. In this paper, the Euclidean distance is employed as the distance measure [[51], [52], [53], [54]].

### 2.3 Fisher Score

The Fisher score holds considerable importance in the realm of reducing high-dimensional data. Its primary objective is to pinpoint a subset of features that maximizes the separation between data points from distinct classes, while simultaneously minimizing the proximity of data points within the same class. This pursuit takes place within the confines of the data space defined by the chosen features. Regarded as a traditional supervised feature selection method, the Fisher score seeks out optimal features that play a dual role: decreasing the spread of data points within individual classes (within-class scatter) and amplifying the differentiation between different classes (between-class scatter). This strategic approach proves valuable in enhancing the effectiveness of classification tasks.

(1) $$S_{j\;}=\frac{\sum n_{j\;}{\left(\mu_{ij\;}-\mu_{i}\right)}^{2}}{\sum n_{j}*{\rho_{ij}}^{2}}$$

Initially, the Fisher Score (F.S.) assigns a score to each facet of the original data set. Subsequently, a subset of the most promising features is derived from these scores. The Fisher score for the Kth feature can be computed independently, and then features with higher Fisher scores are chosen for inclusion [55,56].

### 2.4 Wilcoxon Rank-Sum test

The Wilcoxon Rank-Sum test stands as a non-parametric analysis technique that operates without the requirement of assuming a normal distribution. This method is particularly suited for the comparison of two independent samples, serving to ascertain if their distributions exhibit significant disparities in terms of central tendency—specifically, whether one sample tends to possess higher values than the other. This can be achieved by using the Mann-Whitney *U* test, sometimes referred to as the Wilcoxon Rank-Sum test. This method is used to determine relevance scores for every gene in high-dimensional datasets in the field of machine learning. Thus, by identifying the gene with the highest score, these scores aid in the process of choosing the most dominating gene [57].

### 2.5 Mood's median test

The Mood's Median Test is a statistical method designed for identifying whether there is significant variation between the medians of several groups. This test is especially helpful when working with data that might not have a normal distribution or when there is insufficient information on the actual distribution. This non-parametric test looks at the medians of the data rather than the means, which makes it resilient in situations when the data is ordinal or skewed. Mood's Median Test is a useful tool in many investigations, including those requiring machine learning and feature comparisons, since it compares medians across groups to assist us identify if there are important differences that retain statistical significance [57,58].

### 2.6 Signal-to-noise ratio

The relative intensity of a desired signal in relation to the background noise in each data set or system is measured using the SNR metric.

Mathematically, SNR is often expressed as:

(2) $$SNR=\frac{signal\;power}{Noise\;Power}$$

where the power of the signal of interest is indicated by signal power. In the same way that noise power indicates the amount of background noise or interference, it shows the strength of significant information or data. Any unwelcome or erratic changes that might obstruct the signal are included.

Better data clarity and dependability are achieved when there is a greater signal-to-noise ratio (SNR) signal, which is more pronounced and easier to discern. A lower SNR, on the other hand, suggests that there is comparatively more noise surrounding the signal, which might make it more difficult to detect or analyze the intended signal. For precise measurements and trustworthy results in study and testing, a high SNR is essential [59,60].

### 2.7 Proposed method

The objective of the proposed method is to perform robust feature or gene selection in high-dimensional datasets by leveraging the Mood Median Test and focusing on outlier elimination, feature selection, and redundant gene elimination, the technique aims to enhance the quality and reliability of analyses performed on complex biological or computational datasets.

The proposed method named Md is based on to noise ratio and the P-value of the Mood, median test, the function is given by.

(3) $$\text{Md}=\frac{SNR}{P\;}$$

Single to-noise ratio value for each gene requires a high score to select the optimal genes or features in the high-dimension data set therefore the proposed method uses Mood's Median test for every gene or feature to find the significance P-value for every gene. The Mood's Median test is the robust statistic test to eliminate the outlier effect and also find the significance value for the best or optimal genes.

When the SNR value is divided by the significance P-value for every gene in the data set. Md value is high for the optimal genes because the SNR value is required high value for the feature or genes and the P-value is required smaller when the genes or feature is significant. When the high value of SNR is divided by the significance P-value then the Md value is high for best or optimal genes. So, after the top highest scores of genes 5, 10, 15, and 20 are selected to find out the classification error of the *KNN* and R.F classifiers**.**

## 3 Result and discussion

To assess the effectiveness of the proposed method Mood Median test is more robust in the single-to-noise Ratio [57]. The UC Irvine Machine Learning Repository has different gene expression datasets but, in this article, three distinct gene expression datasets are employed: lung cancer data, A.P.- Breast -Prostate data, and the DLBCL data [59,60]. Comprehensive details regarding each dataset can be found in Table 1.

**Table 1:** Comprehensive details of datasets.

Datasets	Samples	Genes	Classes
AP-Breast-Prostate	413	10937	2
DLBCL	76	5470	2
Lung Cancer	148	12534	2

When computing the similarity parameters, the Mood Median test is used to identify statistically significant genes, and the SNR score is used to quantify the category importance of the genes. Next, based on these characteristics, the RF and KNN algorithms assess their performance in gene categorization tasks [49,51,52]. The high dimension datasets come to have UC Irvine Machine Learning Repository with binary classes. A.P. Breast -Prostate data set is the binary class data in this data set the number of observations is 413 and the number of genes or features is 10937. The DLBCL data set contains 5470 features or genes, which is more than the 76 samples or observations. The lung cancer binary classes data set includes 148 samples and 12534 genes or traits. In the high-dimension data set, there are some redundant features or genes these features have not contributed to the binary classes. In the case of feature selection used robust method to select the optimal genes or features [[55], [56], [57]].

When applied to different gene expression datasets, the hybrid method (MD) shows remarkable resilience and outperforms other feature selection approaches including Fisher, Wilcoxon, and SNR methods [[61], [62], [63]]. When used with machine learning algorithms such as K-Nearest Neighbors (K-NN), Random Forest, and Support Vector Machine (SVM), it achieves significantly reduced classification error rates, demonstrating its superiority [[37], [38], [39]]. Due to its sophisticated statistical measure integration, the MD approach improves the performance of these classifiers by more precisely identifying the most relevant characteristics. As a result, in high-dimensional gene expression analysis, it turns out to be a more dependable and successful feature selection technique [[64], [65], [66]].

The preceding tables were utilized to determine the classification error rates within a high-dimensional dataset. Table 1 pertains to the AP-Breast-Prostate dataset, where the Fisher method demonstrated the lowest classification error rates for the top 5, 10, 15, and 20 genes or features. Notably, the classification error rate based on the SNR is minimal for genes 5 and 15, whereas other method-based error rates exceed those of the Fisher method [[55], [56], [57]]. Consequently, the Fisher method showcased superior performance on the AP-Breast-Prostate dataset.

Table 2 pertains to the DLBCL dataset, where the proposed method Md(function) exhibited the lowest classification error rate compared to other methods. Notably, for all variations of the top 5, 10, 15, and 20 genes or features, the Random Forest and K-Nearest Neighbors classifiers demonstrated the minimum classification error rates.

**Table 2:** Average classification error rates for AP-Breast-Prostate data set based on Random Forest (R.F.) and K-Nearest Neighbors (*K-NN*), Random Forest.

Random Forest				
Genes	Md	Fisher	Wilcox	SNR
5	0.100	0.009	0.019	0.009
10	0.007	0.005	0.010	0.006
15	0.009	0.007	0.010	0.007
20	0.005	0.004	0.008	0.005
K-Nearest Neighbors (*K*-NN)				
Genes	Md	Fisher	Wilcox	SNR
5	0.135	0.011	0.020	0.010
10	0.012	0.009	0.011	0.008
15	0.015	0.010	0.015	0.010
20	0.014	0.008	0.017	0.007

In Table 3, which represents the lung cancer dataset, the proposed method demonstrated superior performance compared to other methods for the top 5, 10, 15, and 20 genes or features.

**Table 3:** Average classification error rates for DLBCL data set based on Random Forest (R.F.) and K-Nearest Neighbors (*KNN*).

Random Forest				
Genes	Md	Fisher	Wilcox	SNR
5	0.000	0.001	0.096	0.003
10	0.001	0.019	0.113	0.024
15	0.020	0.050	0.114	0.093
20	0.010	0.051	0.108	0.050
K-Nearest Neighbors (CNN)				
Genes	Md	Fisher	Wilcox	SNR
5	0.016	0.016	0.024	0.010
10	0.004	0.008	0.019	0.012
15	0.003	0.009	0.011	0.008
20	0.009	0.010	0.016	0.084

Our proposed approach, which is based on Mood's test, aims to calculate P-values for each gene using three distinct metrics, SNR score, Fisher score, and the Wilcox method. This study analyzes three high-dimensional datasets to identify the most valuable genes or features. Our primary objective is to eliminate redundant genes or features, as these do not contribute significantly to the response groups. In both the DLBCL and lung cancer datasets, our proposed method consistently exhibits the lowest classification error rates when compared to three alternative methods: Fisher, Wilcox, and SNR. This study assessed performance using both Random Forest and K-Nearest Neighbors (K-NN) classifiers. In the DLBCL dataset, the classification error rate is minimized when employing the Random Forest and K-NN classifiers, surpassing the performance of the Fisher, Wilcox, and SNR methods.

Similarly, in the case of the lung cancer dataset, our method for gene selection demonstrates the lowest classification error rate among all methods considered. On the other hand, in the Ap Prostate dataset, the Fisher score method outperforms the other methods when utilizing the Random Forest classifier, while the SNR method outshines the rest when employing the K-NN classifier.

In summary, our proposed technique, relying on Mood's test and various evaluation metrics, proves effective in selecting optimal genes or features, particularly in the DLBCL and lung cancer datasets, resulting in superior classification performance compared to alternative methods across different classifiers.

**Fig. 1:**
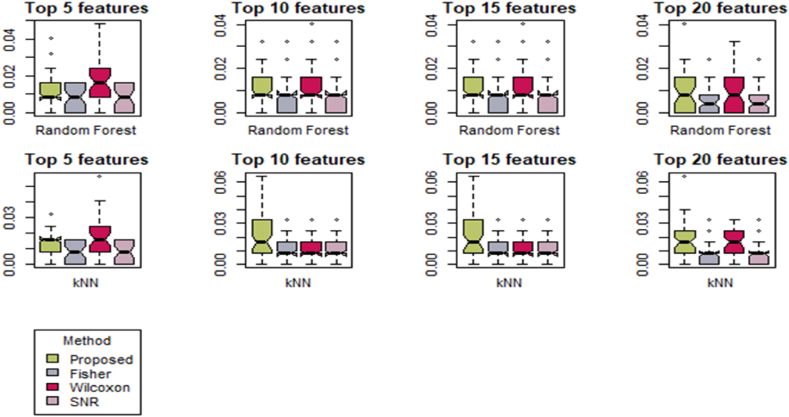
Box plot of AP-Breast-Prostate data set based on Random Forest (R.F.) and K-Nearest Neighbors (*K-NN*) for various feature.

Fig. 1 evaluates the box plot of the AP-Breast-Prostate data set based on Random Forest (R.F.) and K-Nearest Neighbors (*K-NN*) for various features. The box plots for various features based on the Random Forest and K-Nearest Neighbors (K-NN) methods appear to be almost identical. This observation suggests that both Random Forest and K-NN methods are producing similar results in terms of their central tendencies, spreads, and potential outliers.

**Fig. 2:**
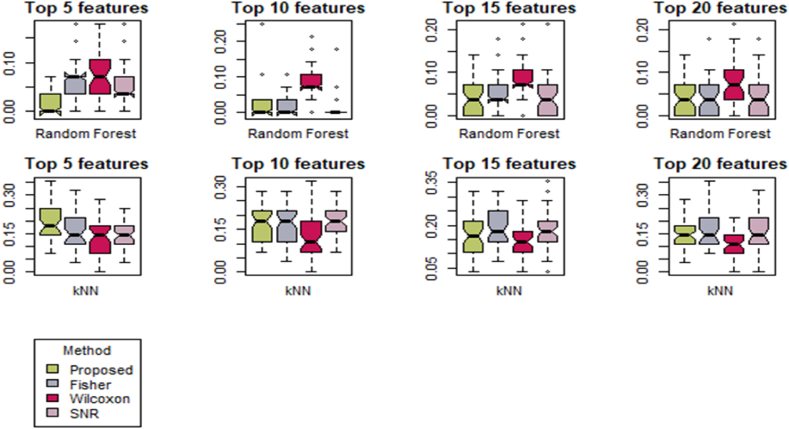
Box plot of DLBCL data set based on Random Forest (R.F.) and K-Nearest Neighbors (*K-NN*) for various feature.

**Fig. 3:**
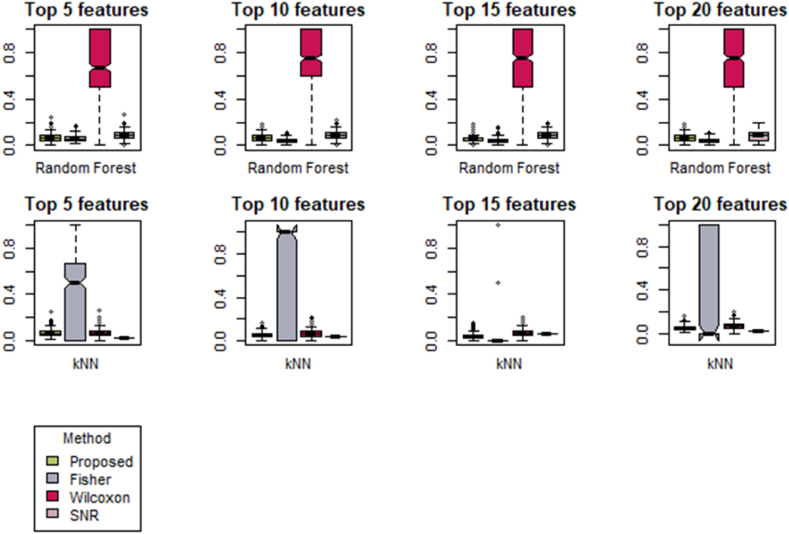
Box plot of the lung data set based on Random Forest (R.F.) and K-Nearest Neighbors (*K-NN*) for various feature.

Fig. 2 displays box plots depicting the AP-Breast-Prostate dataset's analysis using two different machine learning methods: Random Forest (R.F.) and K-Nearest Neighbors (K-NN), across various features. The visual representation reveals that outliers have been identified when employing the Random Forest technique, whereas there are no outliers detected when using the K-NN approach for all four feature sets. Fig. 3 illustrates the box plot of the lung data set for various features based on Random Forest (R.F.) and K-Nearest Neighbors (*K-NN*). The result assesses that the Wilcoxson method has the highest variability in scores and is potentially positively skewed for various features of the Random Forest method. Similarly, the Fisher method explained maximum variability for 5, 10, and 20 features based on K-Nearest Neighbors (*K-NN*) [67,68].

## 4 Conclusion

The central focus of the paper is to identify the optimal genes through a robust hybrid approach, which combines the Mood Median test and the SNR score. The Mood Median test, specifically suited for data that is non-normally distributed or skewed, serves the purpose of pinpointing genes that exhibit significant differences among various groups. On the other hand, the Single Noise Ratio score assesses the classification relevance of individual genes by quantifying the degree of separation between class means relative to within-class variability.

The classification error rates in high-dimensional genomic datasets were assessed using the AP-Breast-Prostate dataset is the subject of Table 4, where the classification error rates for the top 5, 10, 15, and 20 genes were found to be the lowest using the Fisher technique. For genes 5 and 15, the Signal-to-Noise Ratio (SNR) technique had the lowest error rates; nevertheless, generally, the other methods' error rates were greater than the Fisher method's, proving the Fisher method's superior performance in this dataset.

**Table 4:** Average classification error rates for lung data set based on Random Forest (R.F.) and K-Nearest Neighbors (*K-NN*).

Random Forest				
Genes	Md	Fisher	Wilcox	SNR
5	0.020	0.014	0.023	0.012
10	0.011	0.021	0.023	0.014
15	0.012	0.012	0.021	0.012
20	0.014	0.017	0.024	0.014
K-Nearest Neighbors (CNN)				
Genes	Md	Fisher	Wilcox	SNR
5	0.046	0.186	0.137	0.147
10	0.083	0.186	0.165	0.165
15	0.023	0.176	0.165	0.163
20	0.024	0.157	0.162	0.158

The suggested Md(function) technique had the lowest classification error rates among all tested methods on the DLBCL dataset. For every variant of the top 5, 10, 15, and 20 genes in dataset, the Random Forest and K-Nearest Neighbors (KNN) classifiers consistently showed the lowest classification error rates.

Through the application of both the Mood Median test and the SNR score to each gene, the intention is to shed light on genes that play a pivotal role in classification tasks. In summary, the proposed technique, relying on Mood's test and various evaluation metrics, proves effective in selecting optimal genes or features, particularly in the DLBCL and lung cancer datasets, resulting in superior classification performance compared to alternative methods across different classifiers. The analysis reveals that incorporating robust measures of dispersion enhances systems that employ such statistics for feature/gene ranking. Moreover, compared to utilizing tens of thousands of genes, which demands substantial computational resources, outstanding classification accuracy can be achieved with a relatively small subset of genes. From box plots between Random Forest and K-NN methods for the AP-Breast-Prostate dataset, suggesting comparable performance. From the box plot of the lung data set it has been suggested that the Wilcoxson method under Random Forest and the Fisher method under K-NN are associated with distinct characteristics in terms of variability and skewness across the selected features.

### 4.1 Limitations of study

The classifier that is used has an impact on how successful the suggested gene selection technique works. It has been noted that various classifiers (e.g., Random Forest vs. K-Nearest Neighbors) may produce varying classification accuracies, indicating that the approach might not be consistently better than other machine learning models.

The study indicates that a limited group of genes can still yield classification accuracy; nevertheless, the process of selecting features can still be computationally challenging, particularly when utilizing several different approaches. This could restrict its use in situations when resources are scarce or real-time.

Not all datasets may meet the Mood Median test's assumptions about non-normality and skewness. The successful application of the SNR score is contingent upon the presumption of clearly distinguished class means, and this may not hold for datasets that exhibit class overlap.

Overfitting is a constant concern because of the high complexity of genomic data, particularly when working with lower sample numbers. Although cross-validation techniques are used in the study, more research with larger datasets is required to evaluate the method's robustness against overfitting.

Instead of delving thoroughly into the biological importance of the selected genes, the study concentrates on statistical techniques for gene selection. Consequently, additional research is needed to determine the finding applicability in clinical or biological settings.

### 4.2 Future Plan

To assess its generalizability and efficacy under different circumstances, the suggested hybrid technique could be used on high-dimensional genomic datasets other than DLBCL and lung cancer datasets. To confirm and improve the approach, future research could examine a wider range of cancer kinds or different diseases.

Incorporating other feature selection strategies, including information sharing or deep learning-based methods, could potentially enhance the choice of genes and accuracy in classification, even if the Mood Median test and SNR score have demonstrated encouraging results thus far.

The efficacy of additional ML classifiers, such as (SVM) or NN, could be investigated in future research to see if they can further lower classification error rates, even though Random Forest and K-Nearest Neighbors were employed in this investigation.

Prospective investigations may concentrate on incorporating significance biologically or pathway evaluation into the process of feature selection. This might make it easier to choose genes by taking into account both their biological significance and statistical relevance, which could result in more findings that are clinically useful.

The viability of implementing the suggested method in real-time in healthcare facilities could be investigated. To improve performance for various datasets and particular classification tasks, further study might involve improving the parameters of the Mood Median test, SNR score, and classifiers.

## Funding

This research received no external funding.

## Data availability statement

The data used in this study is available at https://archive.ics.uci.edu/.

## CRediT authorship contribution statement

**Ibrar Hussain:** Software, Formal analysis, Data curation, Conceptualization. **Moiz Qureshi:** Resources, Project administration, Methodology, Investigation, Formal analysis, Data curation. **Muhammad Ismail:** Writing – original draft, Resources, Project administration, Methodology, Formal analysis. **Hasnain Iftikhar:** Writing – review & editing, Visualization, Supervision, Project administration. **Justyna Zywiołek:** Writing – review & editing, Validation, Resources, Project administration, Funding acquisition. **Javier Linkolk López-Gonzales:** Writing – review & editing, Supervision, Project administration, Investigation.

## Declaration of competing interest

The authors declare that they have no known competing financial interests or personal relationships that could have appeared to influence the work reported in this paper.
